# The Crude Polysaccharide Derived from *Agaricus subrufescens* Alleviates Alcoholic Liver Injury

**DOI:** 10.3390/foods15071242

**Published:** 2026-04-05

**Authors:** Ziyi Wang, Shien Wang, Jiazhang Bao, Dan Yan, Mei Hu, Xingsheng Lin, Xucong Lv, Penghu Liu

**Affiliations:** 1 College of Life Sciences, Fujian Agriculture and Forestry University, 15 Shangxiadian Road, Fuzhou 350002, China; zyiwang1997@163.com (Z.W.);; 2 National Engineering Research Center of JUNCAO Technology, Fujian Agriculture and Forestry University, 15 Shangxiadian Road, Fuzhou 350002, China; 3 International College of Juncao Science, Fujian Agriculture and Forestry University, 15 Shangxiadian Road, Fuzhou 350002, China; 4 Institute of Food Science and Technology, College of Biological Science and Technology, Fuzhou University, Fuzhou 350108, China

**Keywords:** *Agaricus subrufescens*, polysaccharide, alcoholic liver injury, biochemical parameters

## Abstract

Alcoholic liver injury (ALI) represents a global public health crisis with limited therapeutic options. Polysaccharides from edible mushrooms have emerged as promising candidates for liver protection due to their multifaceted biological activities and low toxicity. A mouse model of ALI was established to investigate the protective effect of *Agaricus subrufescens* polysaccharide on liver injury. The polysaccharide exhibited a non-triple-helix structural, characterized by a rough surface morphology, crack-like features, and a wavy strip structure. The body growth, liver index, serum and liver biochemical parameters, hepatic histopathological characteristics, and hepatic mRNA levels were investigated. The results demonstrated that *A. subrufescens* polysaccharide significantly alleviated liver injury, decreased serum levels of ALT by 36.22% and AST by 31.65%, lowered hepatic MDA content by 33.19%, and increased the activities of antioxidant enzymes, including SOD, GSH-P_X_, and Cat by 12.04%, 9.76% and 18.45%, respectively. Meanwhile, the polysaccharide also regulated the mRNA expression of key genes involved in fatty acid metabolism, oxidative stress, and inflammatory responses. These findings provide theoretical evidence for the efficacy of *A. subrufescens* polysaccharide against alcohol-induced liver injury.

## 1. Introduction

Alcoholic liver injury (ALI), which includes alcoholic hepatitis, hepatic fibrosis, and cirrhosis caused by long-term alcohol intake, continues to be a significant global public health issue and a primary factor contributing to liver-associated morbidity and mortality [1]. In addition to direct hepatic damage, ALI is frequently accompanied by systemic metabolic disorders, including dyslipidemia, hypertension, and atherosclerotic changes [2]. Mounting evidence suggests that oxidative stress and inflammatory reactions induced by alcohol play a crucial role in the onset and progression of ALI [3]. Consequently, interventions based on antioxidants have gained growing attention as potential approaches for the prevention and treatment of ALI.

Natural bioactive compounds, particularly polysaccharides, flavonoids, and peptides, have been widely reported to exert hepatoprotective effects by alleviating oxidative stress and inflammatory injury induced by excessive alcohol intake [4]. Among them, polysaccharides represent a class of macromolecules with diverse biological activities, including antioxidant, anti-inflammatory, and immunomodulatory properties [5]. Some polysaccharides isolated from *Angelica sinensis*, *Enteromorpha prolifera*, and Dendrobium officinale have been shown to exhibit anti-ALI activity [6]. Sea buckthorn polysaccharides attenuated ethanol-induced cellular damage by inhibiting inflammatory factor secretion, enhancing antioxidant capacity, and preserving cellular structural integrity [7].

*Agaricus subrufescens*, alternatively referred to as *Agaricus blazei* or *Agaricus brasiliensis*, is regarded as one of the most important mushrooms for both culinary and medicinal purposes in Brazil, East Asia, and Europe [8]. Numerous studies have documented its pharmacological activities, including antitumor, antiviral, anti-inflammatory, antioxidant, and immunomodulatory activities [9]. Polysaccharides are recognized as the primary bioactive constituents of *A. subrufescens* and have been demonstrated to exert potent antioxidant and anti-inflammatory effects [10]. *A. subrufescens* polysaccharide, a typical heteropolysaccharide mainly composed of glucose, arabinose, and mannose, exerts its pharmacological activities mainly owing to its β-glucan components [11,12]. Zhang et al. demonstrated that *A. subrufescens* polysaccharide effectively attenuates DSS-induced colitis by suppressing inflammatory cytokine production, mitigating oxidative stress, and preserving colonic barrier integrity [13]. Li et al. reported that an acidic glucan isolated from *A. subrufescens* exhibits hypolipidemic effects by activating the PPARγ/LXRα/ABCA1/ABCG1 cholesterol metabolic pathway [14]. Despite its widespread dietary use and medicinal potential, the hepatoprotective effects of *A. subrufescens*-derived polysaccharides against alcohol-induced liver injury have not been systematically investigated.

Therefore, the present study aimed to evaluate the protective effects of polysaccharides extracted from *A. subrufescens* on ALI in mice. The effects on body weight, liver index, serum and hepatic biochemical parameters, histopathological changes, and hepatic gene expression were comprehensively assessed. This research offers experimental evidence that supports the potential application of *A. subrufescens* polysaccharides as functional food ingredients or therapeutic agents in the prevention and treatment of ALI.

## 2. Materials and Methods

### 2.1. Materials

The chemical reagents were provided by Sinopharm Chemical Co., Ltd. (Shanghai, China). The serum and liver biochemical kits were all purchased from Nanjing Jiancheng Bioengineering Institute (Nanjing, China). The fluorescence quantitative PCR kits were all from EKERE Biotechnology (Changsha, China). Fifty-two SPF-grade Kunming mice (male, weighing 39 ± 2.1 g) were acquired from Wushi Experimental Animal Trading Co., Ltd. (Fuzhou, China).

### 2.2. Extraction

*A. subrufescens* were harvested from Fujian Agriculture and Forestry University. Dried fruiting bodies of *A. subrufescens* were ground into a fine powder. Boiling water was used to extract the polysaccharides at a solid-to-liquid ratio of 1:30 (g/mL) at 100 °C for 2 h. The extract was filtered, concentrated to 1/5 of its original volume, and deproteinized with a chloroform-isoamyl alcohol (4:1, *v*/*v*) at a volume equivalent to 1/3 of the concentrate. After removing organic solvents through rotary evaporation (R205B, Xiamen Jingyi Xingye Technology Co., Ltd., Xiamen, China), crude polysaccharides were precipitated with four volumes of absolute ethanol at 4 °C for 12 h; subsequent centrifugation was performed at 4000 rpm for a duration of 10 min, followed by freeze-drying. The extraction yield of polysaccharide (*w*/*w*) was 5.63%, and its purity reached 61.1%.

### 2.3. Fourier Transform Infrared Spectroscopy (FTIR) Analysis

The polysaccharide was mixed with KBr at a mass ratio of 1:200, it was pressed into pellets, and it was analyzed using a Thermo Fisher Scientific 6700 FTIR spectrophotometer (Thermo Fisher Scientific, Waltham, MA, USA) over the wavelength range of 400–4000 cm^−1^ [15].

### 2.4. Congo Red Analysis

The polysaccharide (5 mg) was dissolved in 80 μM Congo red reagent, and 1 M NaOH solution was added dropwise to final concentrations ranging from 0 to 0.5 M. The absorption spectra of the mixtures were measured in the range 400–600 nm using a microplate reader (Molecular Devices, SpectraMax, Sunnyvale, CA, USA), with Congo red solution without the polysaccharide as the control. This method was employed to characterize the triple-helix conformation of the polysaccharide.

### 2.5. Scanning Electron Microscope (SEM) Analysis

The surface morphology of polysaccharide was observed using a field-emission SEM (Merlin, Carl Zeiss AG, Oberkochen, Germany) under high vacuum. Dried polysaccharide powder was fixed on a sample stub with double-sided adhesive tape, sputter-coated with a thin gold film, and imaged at an acceleration voltage of 5.0 kV.

### 2.6. Animal Experiments

During the 10-day adaptation period, under controlled environmental conditions (temperature: 24 ± 1 °C, normal day–night cycle, humidity: 60 ± 5%), the mice were housed and provided with a basal diet and water ad libitum. Animal experimental procedures for this study were approved by the Ethics Committee of Institute of Food Science and Technology, Fuzhou University, China (approval no.: FZU-IFST-2021012). Randomization was employed to assign the experimental units to control and treatment groups. A simple random allocation sequence was generated using a random number generator. Afterward, the mice were randomly divided into four equal groups, namely the control group, model group, low-dose polysaccharide (PL) group, and high-dose polysaccharide (PH) group.

Based on previous studies, the PL group and PH group received polysaccharide via gavage at daily doses of 100 mg/kg and 400 mg/kg, respectively [16]. Distilled water was used to resuspend the polysaccharide powder, which was then orally administered at 10:00 a.m. every day for 6 weeks. 50% ethanol was administered to the model group, PL group, and PH group at a dose of 7.5 mL per kg of body weight at 2:00 p.m. every day. During the experiment, all mice were provided with adequate feed and water ad libitum, and their body weights were measured weekly. During the 6-week administration of 50% ethanol, no obvious abnormal symptoms, severe suffering, or unexpected weight loss were observed in the mice, and all animals remained in generally good condition. At the end of the experiment, blood samples and liver tissues were collected and preserved at −80 °C for subsequent analysis.

### 2.7. Determination of Body Weight and Liver Index

During the experimental period, body weights were recorded every week and at sacrifice. The calculation method for the liver index was as follows:

$$\mathrm{liver}\;\mathrm{index}=\frac{\mathrm{liver}\;\mathrm{weight}}{\mathrm{final}\;\mathrm{body}\;\mathrm{weight}}\times 100\%$$

### 2.8. Serum and Liver Biochemical Analysis

Serum levels of total cholesterol (TC), triglycerides (TG), high-density lipoprotein cholesterol (HDL-C), low-density lipoprotein cholesterol (LDL-C), aspartate aminotransferase (AST), and alanine aminotransferase (ALT) were determined using commercial assay kits. Liver levels of TC, TG, catalase (Cat), glutathione (GSH), glutathione peroxidase (GSH-P_X_), superoxide dismutase (SOD), malondialdehyde (MDA), alcohol dehydrogenase (ADH), and aldehyde dehydrogenase (ALDH) were determined using commercial assay kits.

### 2.9. Histopathological Examination of Liver

A ratio of 1%:4% (*w*/*v*) paraformaldehyde was used to fix liver tissues for 24 h, followed by dehydration via a graded ethanol series, paraffin embedding, and sectioning into 5 μm slices. Hematoxylin and eosin (H&E) staining was performed on the sections, which were then observed under a light microscope (Olympus Corporation, Tokyo, Japan). All histological sections were examined in a blinded fashion by an independent pathologist to ensure objective evaluation. Histological evaluation was performed via a semi-quantitative approach based on the integrity of hepatic lobule and hepatocyte cord arrangement, the extent of hepatocellular cloudy swelling (ranging from no swelling with clear cytoplasm and distinct cell boundaries to marked swelling with highly turbid cytoplasm and disordered lobular structure), the degree of hepatocellular foamy change (from no vacuolization to extensive intracellular lipid vacuole accumulation), and the clarity of hepatic nuclear morphology (from round, centrally located nuclei with distinct nucleoli to indistinct, displaced or degenerated nuclei) in liver tissues.

### 2.10. RT-qPCR Analysis

Trizol reagent was used to extract the total RNA from the liver, followed by reverse transcription into cDNA using a commercial cDNA kit equipped with gDNA eraser. RT-qPCR was performed on a StepOne Real-Time PCR system (Applied Biosystems, Foster City, CA, USA) using SYBR^®^ Green Pro Taq HS kit (Accurate Biotechnology, Changsha, China). The PCR program was as follows: the initial denaturation at 95 °C for 30 s, followed by 40 cycles of denaturation at 95 °C for 5 s, annealing at 55 °C for 15 s, and extension at 72 °C for 15 s. β-Actin was used as the internal reference gene, and relative mRNA expression levels were calculated using the 2^−ΔΔCt^ method. Primer sequences are listed in Table 1.

### 2.11. Statistical Analysis

One-way analysis of variance (ANOVA), followed by the LSD post hoc test, was used to determine statistical significance, and all analyses were performed using the GraphPad Prism 7.0 software. The data were presented as mean ± SD, and statistical significance was indicated as ^##^ *p* < 0.01 and ^#^ *p* < 0.05, vs. the model group; ** *p* < 0.01 and * *p* < 0.05, vs. the control group.

## 3. Results

### 3.1. Structural Characterization of A. subrufescens Polysaccharide

**Figure 1:**
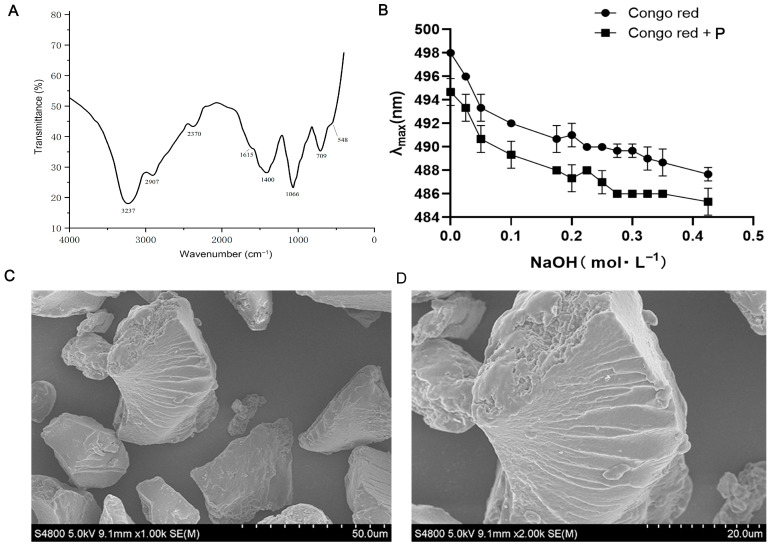
The FTIR spectrum (**A**), Congo red, (**B**) and SEM (**C**,**D**) of *A. subrufescens* polysaccharide.

The structural features of the polysaccharide extracted from *A. subrufescens* were characterized by FTIR spectroscopy, Congo red assay, and SEM analysis. As shown in Figure 1A, the FTIR spectrum exhibited typical polysaccharide absorption bands. At 3200–3600 cm^−1^, a broad and intense absorption peak was observed, which corresponded to O–H stretching vibrations, and the peak at 2907 cm^−1^ was assigned to C–H stretching. At 1615 cm^−1^ and 1400 cm^−1^, absorption bands were detected, which corresponded to the asymmetric stretching vibration of C=O groups and C–H (or O–H) bending vibrations, respectively.

The Congo red assay is performed to evaluate the presence of a triple-helix conformation. As shown in Figure 1B, at 0 mol·L^−1^ NaOH, the maximum absorption wavelength (λmax) of the Congo red control was 498 nm, while that of the polysaccharide–Congo red complex (Congo red + P) was 495 nm. With the increase in NaOH concentration, no obvious red shift was found in the maximum absorption wavelength of the polysaccharide–Congo red complex, indicating that the polysaccharide did not possess a triple-helix structure. The surface structure of the *A. subrufescens* polysaccharide was further examined through SEM (Figure 1C,D). The polysaccharide particles exhibited an irregular granular morphology with a rough surface, characterized by crack-like structures and wavy strips, indicating a loose and heterogeneous microstructure.

### 3.2. Effect of A. subrufescens Polysaccharide on Body Weight and Liver Index in ALI Mice

**Figure 2:**
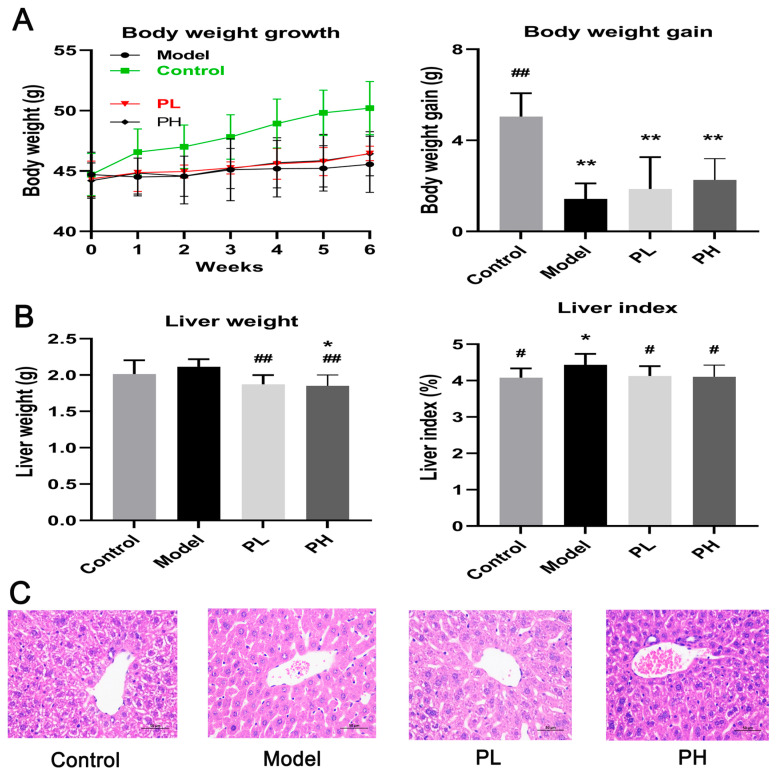
The effects of *A. subrufescens* polysaccharide treatment on the body weight growth in ALI mice (**A**). The effects of *A. subrufescens* polysaccharide treatment on the liver index in ALI mice (**B**). The effects of *A. subrufescens* polysaccharide on the liver histological morphology of ALI mice (**C**). Note: ^##^ *p* < 0.01 and ^#^ *p* < 0.05, vs. the model group; ** *p* < 0.01 and * *p* < 0.05, vs. the control group.

As shown in Figure 2A, no differences were observed in the body weights of mice among the experimental groups at the start of the experiment. The body weights of mice in all groups showed a tendency to increase with the extension of the test days. After 6 weeks of intragastric administration of 50% alcohol, the body weight of mice in the model group was significantly lower than that in the control group (*p* < 0.05), suggesting that excessive alcohol consumption may impair metabolic function. The administration of *A. subrufescens* polysaccharides alleviated alcohol-induced body weight loss, and the effect tended to be enhanced with increasing dosage, with the PH group exhibiting a more notable protective effect. Additionally, the liver index can be used to a certain extent effectively reflect the degree of liver damage (Figure 2B). The liver index of mice in the model group was significantly higher than that in the control group (*p* < 0.05), whereas high-dose polysaccharide treatment significantly decreased the liver index compared with the model group (*p* < 0.05).

### 3.3. Effect of A. subrufescens Polysaccharide on Histopathological Features of Liver in ALI Mice

The histopathological alterations in the liver tissues of mice across various experimental groups were presented in Figure 2C. The mice in the control group exhibited distinct arrangements of liver lobules and hepatocyte cords, with round central nuclei, clear cytoplasm, and distinct cell boundaries. In contrast, excessive alcohol consumption resulted in obvious hepatocyte cloudy swelling and indistinct nuclei, which suggested the accumulation of lipids in intracellular vesicles. As shown in Figure 2C, the hepatocyte swelling and foam-like changes induced by excessive alcohol intake were alleviated in the group treated with PL and PH. The symptoms were significantly relieved in the mice treated with high-dose polysaccharide, which was similar to the control group without alcohol.

### 3.4. Effect of A. subrufescens Polysaccharide on Serum Biochemical Parameters in ALI Mice

**Figure 3:**
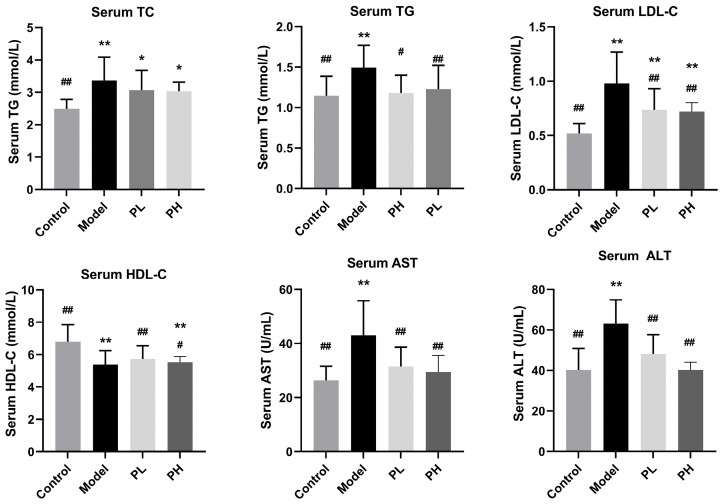
The effects of *A. subrufescens* polysaccharide treatment on the serum biochemical parameters of ALI mice. Note: ^##^ *p* < 0.01 and ^#^ *p* < 0.05, vs. the model group; ** *p* < 0.01 and * *p* < 0.05, vs. the control group.

As useful and important diagnostic indicators, serum biochemical indexes can be applied in the diagnosis of ALI. Compared with the control group, there was a significant increase in the serum TC, TG, and LDL-C levels in the model group, and a significant decrease in the serum HDL-C level, which suggests that the ALI model had been successfully constructed (Figure 3). In contrast, significant changes in TG, LDL-C, and HDL-C levels were observed in the PH group compared with the model group. Specifically, compared with the model group, PH treatment decreased TC, TG, and LDL-C levels by 10.40%, 21.09%, and 26.66%, respectively, while significantly increasing HDL-C levels by 2.85%, indicating that *A. subrufescens* polysaccharide effectively preserved the normal cholesterol transport pathway and enhanced lipid metabolism. In addition, as the two most important markers of liver metabolic function, serum AST and ALT activities were significantly increased in mice exposed to excessive alcohol consumption. As shown in Figure 3, the results of the study indicated that oral supplement of *A. subrufescens* polysaccharide significantly ameliorated alcohol-induced abnormal increases in serum AST and ALT (*p* < 0.01). Compared with the model group, PH treatment reduced AST and ALT levels by approximately 31.65% and 36.22%, respectively, implying that *A. subrufescens* polysaccharide could effectively ameliorate liver injury. And the results suggested that high-dose polysaccharide could effectively alleviate alcohol-induced liver metabolic disorders.

### 3.5. Effect of A. subrufescens Polysaccharide on Liver Biochemical Parameters in ALI Mice

**Figure 4:**
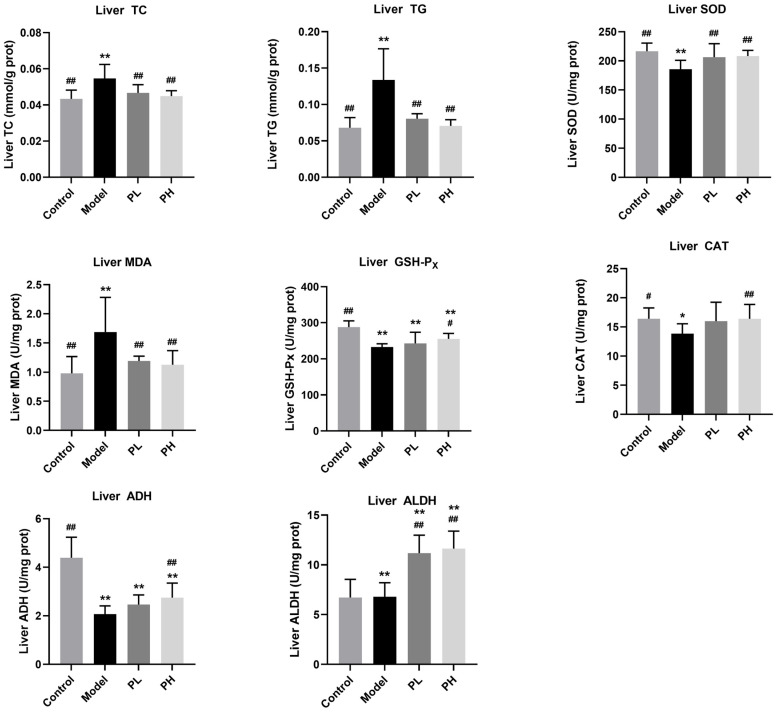
The effects of *A. subrufescens* polysaccharide treatment on the liver biochemical parameters of ALI mice. Note: ^##^ *p* < 0.01 and ^#^ *p* < 0.05, vs. the model group; ** *p* < 0.01 and * *p* < 0.05, vs. the control group.

One of the major organs involved in alcohol metabolism is the liver, and long-term excessive alcohol intake may cause abnormal increases in liver TC and TG levels, which in turn leads to impaired liver metabolic function. As shown in Figure 4, the liver TC and TG levels in the model group mice were significantly higher than those in other groups, indicating that the cholesterol transport pathway in ALI mice was impaired and lipid metabolism was disrupted. After 6 weeks of *A. subrufescens* polysaccharide administration, alcohol-exposed mice showed a significant inhibition of hepatic TC and TG levels. Compared with the model group, PH treatment reduced hepatic TC and TG levels by approximately 17.99% and 47.29%, respectively, exhibiting better therapeutic efficacy. The status of oxidative stress can reflect the extent of liver damage induced by excessive alcohol consumption, and we also detected the levels of oxidative stress-associated parameters (SOD, MDA, GSH-P_X_, and Cat) in the liver. As expected, high-dose polysaccharide significantly reversed the decrease in antioxidants (GSH-P_X_, Cat, and SOD) and alcohol-metabolizing enzymes (ADH, ALDH) levels, and the increase in lipid oxidation indicator (MDA). Compared with the control group, GSH-P_X_, SOD and ADH activities in the PH group were restored to approximately 88.65%, 96.11%, and 62.61%, respectively.

In summary, *A. subrufescens* polysaccharide had a multifaceted protective effect on the liver, including enhancing antioxidant activity, increasing the activity of alcohol-metabolizing enzymes, and reducing oxidative stress, and all of these effects exhibited a dose-dependent trend.

### 3.6. Effect of A. subrufescens Polysaccharide on Hepatic mRNA Expression Levels in ALI Mice

**Figure 5:**
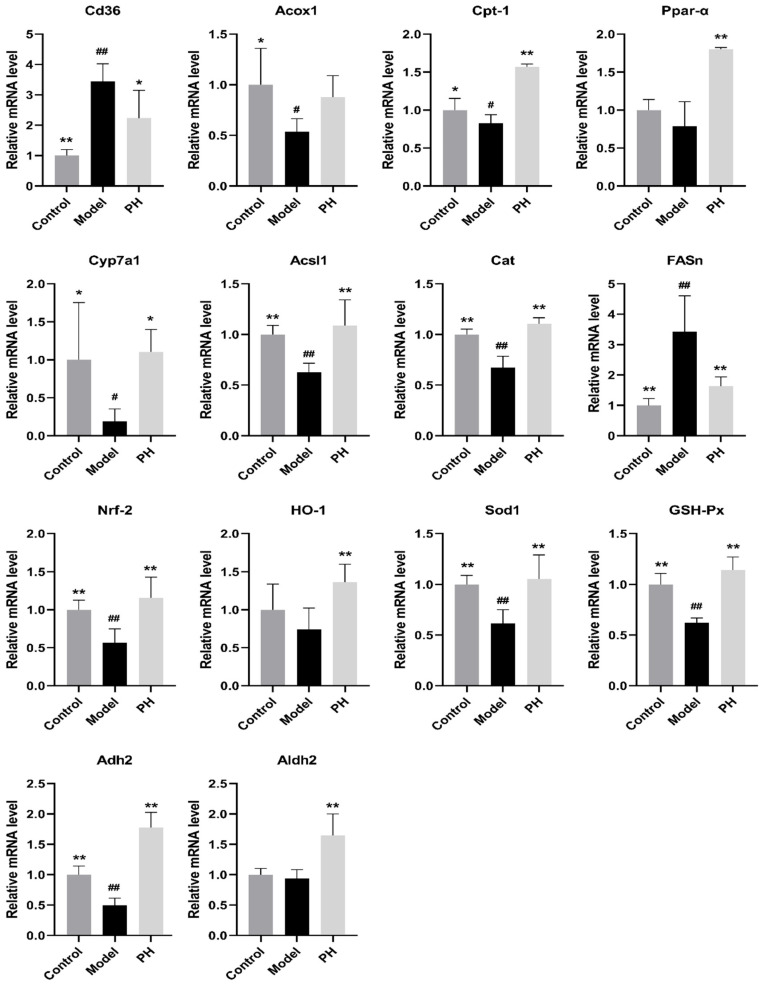
The effects of *A. subrufescens* polysaccharide treatment on the hepatic mRNA expression levels in the livers of ALI mice. Note: ^##^ *p* < 0.01 and ^#^ *p* < 0.05, vs. the model group; ** *p* < 0.01 and * *p* < 0.05, vs. the control group.

To clarify the potential mechanism through which *A. subrufescens* polysaccharide intervention protects against alcohol-induced liver damage, we further analyzed the mRNA expression levels of alcohol metabolism-related genes in the liver via RT-qPCR (Figure 5). Genes related to lipid metabolism investigated in this study included the cluster of Cd36 molecule (*Cd36*), acyl-CoA oxidase 1 (*Acox1*), carnitine palmitoyltransferase1 (*Cpt-1*), peroxisome proliferator-activated receptor α (*Ppar-α*), cholesterol-7α-hydroxylase (*Cyp7a1*), acyl-CoA synthetase long-chain family member 1 (*Acsl1*), catalase (*Cat*), and fatty acid synthase (*Fasn*). In the model group, the mRNA levels of *Acox1*, *Acsl1*, *Cat*, *Cyp7a1*, *Ppar-α*, and *Cpt-1* were significantly downregulated, and the mRNA levels of *Cd36*, *Fasn* were significantly upregulated, which suggested that excessive alcohol intake can impact the lipid metabolism and provoke hepatic cholesterol accumulation. However, the PH group significantly reversed the abnormal expression of the aforementioned genes, suggesting that *A. subrufescens* polysaccharides can improve lipid metabolism. The genes related to oxidative stress were investigated in this study, including the genes of nuclear factor, erythroid 2-like 2 (*Nrf2*), heme oxygenase-1 (*HO-1*), superoxide dismutase-1 (*Sod1*), and glutathione peroxidase (*GSH-P_X_*). Compared with healthy mice, the transcription levels of genes associated with oxidative stress and antioxidant defense in the alcohol group were significantly decreased. The mRNA levels of *Nrf2*, *HO-1*, *Sod1*, and *GSH-P_X_x* were found to significantly increase after high-dose polysaccharide intervention. Notably, the upregulation of the Nrf2/HO-1 signaling pathway effectively mitigates hepatic oxidative stress and strengthens the endogenous antioxidant defense system. It indicates that *A. subrufescens* polysaccharide was capable of suppressing oxidative stress damage triggered by alcohol metabolism. ADH2 and ALDH2 are important enzyme activities associated with alcohol metabolism, the mRNA expression levels of *Adh2* and *Aldh2* were decreased in the alcohol group. The intervention of high-dose polysaccharide was found to upregulate the mRNA level of *Adh2* and *Aldh2*, which can facilitate the metabolism of alcohol. These indicated that *A. subrufescens* polysaccharide may help strengthen the hepatic antioxidant defense system and alleviate oxidative stress caused by heavy alcohol consumption.

## 4. Discussion

Globally, an estimated 200 million adults consume alcohol routinely, with heavy alcohol intake responsible for roughly 3 million deaths, equivalent to 6% of total deaths [17]. At present, the main strategies for the management of ALI consist of alcohol abstinence, nutritional support, and pharmacological treatment. Currently, drug-based interventions for the prevention and alleviation of ALI have shown certain beneficial effects [18]. However, the development of pharmacotherapies remains relatively limited. Moreover, these agents may aggravate the metabolic burden of the injured liver and cause severe adverse reactions [19]. Natural polysaccharides have been reported to exert favorable protective effects against liver diseases, yet their underlying molecular mechanisms remain to be fully elucidated [16].

Polysaccharides, as natural products, are widely utilized as dietary supplements and therapeutic agents for the prevention of metabolism-related disorders. Notably, polysaccharides derived from fungi have been demonstrated to possess antioxidant and hepatoprotective effects in acute alcohol-induced mouse models [20]. A water-insoluble polysaccharide isolated from *Wolfporia* cocos has been demonstrated to activate the PPAR-γ pathway, thus alleviating inflammatory responses, improving liver injury, and lowering lipid deposition in mice with alcohol-induced hepatic steatosis [21]. In addition, a novel polysaccharide isolated from *Coriolus versicolor* has been reported to exert significant hepatoprotective effects by attenuating oxidative stress and modulating immune function [22]. *W. cocos* polysaccharides are typically characterized by a triple-helical conformation, whereas the ASP investigated herein does not form a triple-helical structure [23]. These structural discrepancies fully highlight the unique properties and specific chemical identity of the *A. subrufescens* extract. These findings on bioactive polysaccharides provide important implications for the development of natural polysaccharides as potential agents against ALI. The prevention and treatment of ALI rely on enhancing anti-inflammatory and antioxidant effects, as well as on reducing hepatic lipid accumulation. Notably, the polysaccharide extract used in this study was a crude fraction with a purity of only 61.1%, and thus the observed hepatoprotective, antioxidant, and lipid metabolism-regulating effects cannot be attributed solely to polysaccharides; other bioactive components present in the crude extract (e.g., small molecular phenolics, peptides, or triterpenoids) may also have contributed synergistically to the observed biological activities.

Polysaccharides have been documented to efficiently scavenge free radicals and exert protective effects. In the present research, the antioxidant activity of *A. subrufescens* polysaccharides in vivo was evaluated. This study observed that the intake of high-dose polysaccharide significantly increased the contents of SOD, GSH, GSH-P_X_, ADH, ALDH, and Cat in mice, improved antioxidant status, and attenuated alcohol-mediated liver damage. After the treatment with *A. subrufescens* polysaccharide, the hepatic MDA level was significantly reduced compared with the model group. As MDA is a key marker of lipid peroxidation, the decreased MDA level directly indicates the alleviation of alcohol-induced lipid peroxidation [24], which helps maintain cellular structural integrity and ameliorates oxidative liver damage. Insufficient activity of ADH and ALDH can lead to the accumulation of acetaldehyde, causing oxidative stress and apoptosis of liver cells [25]. The intervention of *A. subrufescens* polysaccharide has been shown to enhance the activity of ADH and ALDH, which can reduce the risk of ALI. Furthermore, chronic alcohol intake impairs the permeability of hepatic cell membranes, leading to marked increases in serum ALT and AST levels, which serve as key biomarkers of liver damage [26]. Serum biochemistry, liver index, histopathological staining, and ultrastructural examination collectively confirmed the successful establishment of the ALI mouse model. Meanwhile, these results demonstrated that *A. subrufescens* polysaccharide exerted effective hepatoprotection against alcohol-induced liver injury. Although histopathological analysis revealed a clear attenuation of alcohol-induced hepatic steatosis, inflammatory infiltration, and necrosis in polysaccharide-treated groups, this analysis was primarily qualitative in nature. This qualitative assessment represents a limitation of the present study, as it lacks the precision of fully quantitative morphological analysis to confirm the degree of histological improvement.

To explore the underlying mechanism of *A. subrufescens* polysaccharide against alcohol-induced liver damage, the mRNA expression of genes associated with alcohol metabolism was further determined through RT-qPCR. *Acsl1*, *Cpt-1*, *Acox1*, *Ppar-α*, and *Cd36* are all key genes involved in the regulation of hepatic lipid metabolism. *Acsl1* catalyzes the synthesis of long-chain acyl-CoA, a precursor for triglyceride biosynthesis, while *Cpt-1* converts the conversion of long-chain fatty acyl-CoA to acylcarnitine, which is involved in fatty acid metabolism. It is well recognized that *Acox1*, *Cpt-1*, and *Ppar-α* are regulators controlling the *β*-oxidation of fatty acids. *Ppar-α*, in particular, can maintain hepatic lipid homeostasis by regulating *Acox1* expression [27]. *Cd36* functions both as a lipid transporter protein and as a pattern recognition receptor on the cell surface. It plays a pivotal role in regulating fatty acid homeostasis, which in turn exerts an influence on the progression of liver steatosis [28]. *Nrf2* and *HO-1* constitute a central signaling axis that governs the hepatic antioxidant defense system and protects against ethanol-induced oxidative damage. *Nrf2* has been widely researched as a therapeutic target for ALI, because its role as a master regulator of the cellular adaptive antioxidant defense to oxidative stress and its ability to facilitates the detoxification of various toxins, *Nrf2* activation induces the expression of *HO-1,* thereby enhancing antioxidant defense [29]. *Adh2* and *Aldh2* are critical rate-limiting enzyme genes responsible for hepatic ethanol metabolism. The abnormal expression of Adh2 and Aldh2 genes in the liver is extremely related to the occurrence of various liver diseases [30]. Interestingly, the results showed that the *A. subrufescens* polysaccharide treatment significantly inhibited the mRNA expression of *Fasn* and *Cd36* but enhanced the mRNA transcription of *Acsl1*, *Cpt-1*, *Adh2*, *Aldh2*, *Nrf2*, *GSH-P_X_*, *Sod1*, and *Cat*. The above results indicated that the *A. subrufescens* polysaccharide treatment modulated the mRNA expression of genes related to hepatic lipid metabolism, antioxidation and inflammation. The present study revealed that *A. subrufescens* polysaccharide modulated a coordinated molecular network rather than individual genes. Specifically, the polysaccharide activated the Nrf2/antioxidant axis to upregulate *Nrf2*, *HO-1*, *Sod1*, *GSH-P*_X_ and *Cat*, thereby reinforcing hepatic antioxidant capacity and alleviating oxidative stress. Meanwhile, it regulated the Ppar-α-mediated lipid metabolism pathway by increasing *Ppar-α*, *Cpt-1*, *Acox1*, and *Acsl1* expression and decreasing *Cd36* and *Fasn* transcription, thus reducing lipid uptake and promoting fatty acid β-oxidation to alleviate hepatic steatosis. In addition, the polysaccharide enhanced ethanol metabolism via upregulating *Adh2* and *Aldh2* to accelerate alcohol clearance and reduce acetaldehyde-induced cytotoxicity. These three pathways interact and complement each other, forming a systematic protective mechanism that ultimately mitigates alcohol-induced liver injury.

## 5. Conclusions

In the current study, an active polysaccharide from *A. subrufescens* was successfully isolated. The polysaccharide exhibited a non-triple-helix structural configuration, characterized by a rough surface morphology, crack-like features, and a wavy strip structure. Critical structural parameters such as monosaccharide composition ratio, glycosidic bond type, molecular weight distribution, and degree of branching were not elucidated in this study. *A. subrufescens* polysaccharides could effectively regulate enzymes related to alcohol metabolism, enhance antioxidant capacity, inhibit oxidative stress and inflammatory damage, regulate lipid metabolism, and reduce lipid accumulation. Notably, this polysaccharide actively modulated the expression of key genes involved in lipid metabolism (*Acsl1*, *Cpt-1*, *Acox1*, *Ppar-α*, and *Cd36*), antioxidant defense (*Nrf2*, *HO-1*), and ethanol metabolism (*Adh2*, *Aldh2*), particularly by regulating the Nrf2/HO-1 antioxidant axis and Ppar-α-mediated fatty acid oxidation pathway, thereby protecting against alcoholic liver injury. Therefore, *A. subrufescens* polysaccharides play a protective role in liver injury caused by ALI, which may promote the development of drugs or functional foods containing natural polysaccharides for hepatic protection. However, this study has several limitations. The structure–activity relationship of the polysaccharide remains unclear, and further purification and structural characterization are needed to identify the key bioactive domains. Future studies should also explore the interaction between *A. subrufescens* polysaccharides and the gut microbiota to determine whether the observed hepatoprotective effects are partially mediated through the gut–liver axis and elucidate the specific structural characteristics of the polysaccharides to clarify their structure–activity relationship with hepatoprotection.

## Figures and Tables

**Table 1:** Primer sequences used for RT-qPCR.

Accession No.	Gene Name	Forward Primer (5′–3′)	Reverse Primer (5′–3′)
NM_015729.4	*Acox1*	GCCTGCTGTGTGGGTATGTCATT	GTCATGGGCGGGTGCAT
NM_007981.5	*Acsl1*	CACTTCTTGCCTCGTTCCAC	GTCGTCCCGCTCTATGACAC
NM_011996.2	*Adh2*	AACGGTGAGAAGTTCCCAAAA	ACGACCCCCAGCCTAATACA
NM_009656.4	*Aldh2*	ATCCTCGGCTACATCAAATCG	GTCTTTTACGTCCCCGAACAC
NM_009804.2	*Cat*	TCACCCACGATATCACCAGA	AGCTGAGCCTGACTCTCCAG
NM_007643.5	*Cd36*	ATGGGCTGTGATCGGAACTG	GTCTTCCCAATAAGCATGTCTCC
NM_013495.2	*Cpt-1*	TCCATGCATACCAAAGTGGA	TGGTAGGAGAGCAGCACCTT
NM_007988.3	*Fasn*	CTGCCACAACTCTGAGGACA	TTCGTACCTCCTTGGCAAAC
NM_008160.6	*GSH-P_X_*	CCACCGTGTATGCCTTCTCC	AGAGAGACGCGACATTCTCAAT
NM_010442.2	*HO-1*	AACAAGCAGAACCCAGTCTATGC	AGGTAGCGGGTATATGCGTGGGCC
NM_007824.3	*Cyp7a1*	CCTTGGGACGTTTTCCTGCT	GCGCTCTTTGATTTAGGAAG
NM_010902.5	*Nrf2*	CCGGGGAACAGAACAGGAAA	ACGTTGTCCCCATTTTTGCG
NM_011144.6	*Ppar-α*	TGCCTTCCCTGTGAACTGAC	TGGGGAGAGAGGACAGATGG
NM_011434.2	*Sod1*	TTGGCCGTACAATGGTGG	CGCAATCCCAATCACTCCAC
NM_007393.5	*β-Actin*	TGTTACCAACTGGGACGACA	GGGGTGTTGAAGGTCTCAAA

## Data Availability

The original contributions presented in this study are included in the article. Further inquiries can be directed to the corresponding authors.
